# 223. The Value of Neutrophil to Lymphocyte Count Ratio for Predicting the Clinical Outcomes of Patients with Carbapenem-resistant *Klebsiella pneumonia* Blood Stream Infection

**DOI:** 10.1093/ofid/ofab466.425

**Published:** 2021-12-04

**Authors:** Heng Wu

**Affiliations:** Department of Infectious Diseases, Sir Run Run Shaw Hospital, College of Medicine, Zhejiang University, Hangzhou, Zhejiang, China

## Abstract

**Background:**

The neutrophil to lymphocyte count ratio (NLR) has been recognized as a useful marker of inflammation. But, the prognostic function of NLR in patients with Carbapenem-resistant *Klebsiella pneumonia* (CRKP) blood stream infection is still largely unknown. The aim of this study was to explore the relationship between postoperative NLR and mortality in those patients.

**Methods:**

We performed a retrospective study based on the database from Computerized Patient Record System in Sir Run Run Shaw Hospital from 1/1/2017 to 31/10/2020. Logistic analysis was performed to assess the associations between NLR and 28-day mortality. Multivariate analyses were used to control for confounders.

**Results:**

A total of 134 CRKP blood stream infection inpatients were included in this study, including 54 fatal cases and 80 survival cases on the 28-day after the onset of CRKP BSI, the overall 28-day mortality rate of patients with a CRKP BSI episode was 40.3% (54/134). We conducted a multivariate analysis on these 134 patients and found that APACHE II score on the 4^th^ day (OR 1.379 95% CI 1.065- 1.785, *p* = 0.015), NLR on the 4^th^ day (OR 1.134 95% CI 1.054- 1.221, *p* = 0.001) were significant risk factors for the 28-day mortality of CRKP BSI patients

**Conclusion:**

Elevated NLR was significantly associated with increased 28-day mortality as well as APACHE II score on the 4^th^ day after first positive culture.NLR is promising to be a readily available and independent prognostic biomarker for patients with CRKP blood stream infection.

**Disclosures:**

**All Authors**: No reported disclosures

